# In search of causality: a systematic review of the relationship between the built environment and physical activity among adults

**DOI:** 10.1186/1479-5868-8-125

**Published:** 2011-11-13

**Authors:** Gavin R McCormack, Alan Shiell

**Affiliations:** 1 Population Health Intervention Research Centre, Department of Community Health Sciences, University of Calgary, Alberta, Canada

**Keywords:** urban form, causation, neighborhood self-selection, walkability, physical activity

## Abstract

**Background:**

Empirical evidence suggests that an association between the built environment and physical activity exists. This evidence is mostly derived from cross-sectional studies that do not account for other causal explanations such as neighborhood self-selection. Experimental and quasi-experimental designs can be used to isolate the effect of the built environment on physical activity, but in their absence, statistical techniques that adjust for neighborhood self-selection can be used with cross-sectional data. Previous reviews examining the built environment-physical activity relationship have not differentiated among findings based on study design. To deal with self-selection, we synthesized evidence regarding the relationship between objective measures of the built environment and physical activity by including in our review: 1) cross-sectional studies that adjust for neighborhood self-selection and 2) quasi-experiments.

**Method:**

In September 2010, we searched for English-language studies on built environments and physical activity from all available years in health, leisure, transportation, social sciences, and geographical databases. Twenty cross-sectional and 13 quasi-experimental studies published between 1996 and 2010 were included in the review.

**Results:**

Most associations between the built environment and physical activity were in the expected direction or null. Land use mix, connectivity and population density and overall neighborhood design were however, important determinants of physical activity. The built environment was more likely to be associated with transportation walking compared with other types of physical activity including recreational walking. Three studies found an attenuation in associations between built environment characteristics and physical activity after accounting for neighborhood self-selection.

**Conclusion:**

More quasi-experiments that examine a broader range of environmental attributes in relation to context-specific physical activity and that measure changes in the built environment, neighborhood preferences and their effect on physical activity are needed.

## Background

Despite the continued effort of health professionals and agencies to encourage people to participate in physical activity many adults are not active enough to achieve optimal health benefits [1,2]. Improved strategies for increasing physical activity at the population level are necessary. In the last 15 years there has been a growing interest into the role of the built environment in supporting physical activity. Compared with other health promotion approaches, the creation of built environments that support physical activity is a sustainable strategy for encouraging people to adopt, or further increase levels of, physical activity. One's neighborhood is especially important as it is here where the majority of physical activity, including walking, is undertaken [3].

Recent land development patterns have increased distances between homes and destinations, lowered population densities, and led to disconnected street patterns, all of which are characteristic of urban sprawl. Urban sprawl is a major societal problem as it is negatively associated with physical activity and health [4-7]. Characteristics such as land use mix and proximity to home, street or network connectivity, population density, pedestrian infrastructure, aesthetics, and safety are independently correlated with physical activities such as walking and cycling [8-10]. Access to a mix of local recreational and non-recreational destinations (e.g., cafes, grocery stores, food stores, other retail, schools, and services) is positively associated with transportation and leisure walking [11-13]. Neighborhoods that have more intersections (i.e., grid-like street pattern) offer direct and alternative routes to destinations supporting walking compared with neighborhoods that have fewer intersections (i.e., curvilinear-like street pattern) [14,15]. Higher population densities are positively associated with physical activity[14,16], probably because of the relation between population density and other environmental attributes - population being needed to make mixed land use economically viable for example. Positive associations between walking for transport and access to and the quality of pedestrian infrastructure such as maintained sidewalks, pedestrian-level lighting and shade, and street furniture, have also been found [12,17]. Similarly, associations between walking and neighborhood aesthetics (greenery, landscaping, diversity, upkeep, cleanliness etc.), personal safety (e.g., graffitti, incivilities) and traffic safety (i.e., vehicle volume, speed, crossing-aids) have also been found, although these findings are less consistent [9,18,19].

The existence of associations between the built environment and physical activity is promising. This evidence continues to inform land use practices, for example through the development and application of environmental design guidelines [20,21]. This evidence however, has been mostly derived from cross-sectional data. Cross-sectional studies thus far have contributed to a better understanding of the plausibility, consistency, coherence (i.e., changes in the built environment associated with other outcomes related to physical activity - driving and obesity), and specificity (i.e., specific attributes associated with specific types of physical activity) of the built environment-physical activity relationship. However, cross-sectional studies by their design do not provide explicit information about temporal precedence and most to date have not included mechanisms to rule out competing explanations of the built environment-physical activity relationship, such as neighborhood self-selection. The selection or choice of neighborhood in which to live is based on economic, social, lifestyle circumstances, and physical activity and transportation preferences. Those inclined to walk for transportation may seek out and eventually reside in neighborhoods that cater to their preferred transportation mode (e.g., transit access, access to local stores and services, pedestrian infrastructure). As a result, the magnitude of any association between the built environment and physical activity that is estimated from cross-sectional studies not accounting for neighborhood self-selection is likely to be inflated. The failure to take into account neighborhood self-selection is a major limitation of the evidence to date[22]. Adjusting cross-sectional data for neighborhood self-selection using methods such as direct questioning, statistical control, instrumental variable analysis, sample selection models, joint discrete choice models, and structural equation models, as well as quasi-experimental study designs may reduce bias in estimated associations between the built environment and physical activity [23].

Many reviews acknowledge neighborhood self-selection as an issue,[10,24,25] and yet few report the results from studies that account for neighborhood self-selection separate from those that have not [23,26]. One exception is a review of transportation studies that found that associations between the built environment and travel behavior via active (i.e., walking and cycling) and passive modes (i.e., driving) existed independent of neighborhood self-selection [26]. Synthesizing evidence that provides information about causation and not just correlation between the built environment and different types of physical activity, and not just active transportation, will assist in developing evidence-based urban planning policies that will encourage more physical activity. The aim of this study was to review quantitative studies examining associations between the built environment and physical activity that either used a 1) statistical design to adjust for neighborhood self-selection or 2) experimental or quasi-experimental design with assessment of change in both the built environment and physical activity.

## Method

### Data sources and search strategy

In September 2010, we searched for English-language studies on built environments and physical activity from all available years in health, leisure, transportation, social sciences, and geographical databases (i.e., PubMed, Medline, Transport Research Information Services-TRIS, UrbanStudies: Environment Complete, PsychInfo, SportDiscus, and LeisureTourism Abstracts). Keyword and phrase searches within titles and abstracts were undertaken. Terms and their variants used to capture physical activity included: physical activity; exercise; inactivity; walking, bicycling, strolling, leisure-time, sports, recreation, active transportation and pedestrian. Terms and their variants used to capture the built environment included: objective environment, spatial, neighborhood, built environment, physical environment, streetscape, urban form, urban planning, walkability, pedestrian-friendly and GIS.

The search across the seven databases provided 10,175 article hits (PubMed = 3147, Medline = 2008, TRIS = 1562, Urban Studies: Environment Complete = 1419, PsychInfo = 1085, SportDiscus = 825, and LeisureTourism Abstracts). From these we removed duplicate references and studies that were not peer reviewed, were off topic, or reported only qualitative data, referred only to non-adult samples, took place in workplace/worksite settings, or were methodological studies, commentaries, editorials, or literature reviews. Literature review reference lists were searched for relevant articles missed in the initial search. This left 538 potentially eligible abstracts, each of which was reviewed independently by both authors (GRM and AS) to confirm which ones met our inclusion criteria: (1) having measured physical activity; (2) using an objective assessment of the built environment, and (3) used a cross-sectional study design with adjustment for neighborhood self-selection or an experimental or quasi-experimental design. Seventy-two of the 538 articles were selected by either or both authors (inter-rater agreement = 92.1%) to undergo full article review.

The authors reviewed the 72 full articles identifying those that: 1) examined physical activity as an outcome which could measured via questionnaire, interview, diary, survey, pedometer, accelerometer, Global Positioning Satellite (GPS) system, heart rate monitor, direct observation, calorimetry, or doubly-labeled water; 2) objective measures of the built environment using geographical information systems, desktop mapping, or audits; 3) measured or estimated the effect of neighborhood self-selection including administering items capturing neighborhood preference or reasons for moving to the neighborhood, instrumental variable analysis, selection models, structural equations models, joint probability models, and propensity score analysis and; 4) estimated an association between the built environment and physical activity adjusted for neighborhood self-selection in the case of cross-sectional studies, or estimated the change in physical activity relative to the change in the built environment in the case of quasi-experiments. Relevant quasi-experiments included those that measured physical activity either before and after neighborhood relocation or before and after an environmental intervention of modification. Our approach was inclusive hence quasi-experiments could include one or multiple groups (i.e., comparison or control) and the behaviors of the same participants need not have been assessed before and after the intervention (i.e., a separate pre-post sample design). Studies involving children aged < 18 years of age or that used only self-reported measures of the built environment were excluded. Thirty-three articles met the criteria and were consequently included in the review after author consensus (initial inter-rater agreement = 92.9%).

### Data extraction

From the 33 articles we extracted, summarized and tabulated the following information: author details; study setting (place and date data collected), study design (including sample recruitment), sample characteristics (gender, age, and socioeconomic status), physical activity variables, objective environment variables, method for neighborhood self-selection adjustment, covariates, associations between the built environment and physical activity independent of neighborhood self-selection, and where the information provided report whether the built environment-physical activity relationship attenuated after adjustment for neighborhood self-selection. We grouped natural experiments with quasi-experiments as they share common characteristics - i.e., non-random allocation of participants into intervention and comparison groups and an estimation of an intervention effect - although we acknowledge that the former traditionally reflects interventions that result from naturally occurring events while the latter reflects interventions that often involve deliberate manipulation [27]. With regard to built environment interventions and people moving neighborhoods, the delineation between what is considered a "natural" versus an "experimental" intervention is not always clear.

## Results

### Study sample characteristics

The majority of studies were undertaken in the U.S. (n = 29), with one study being conducted each in Canada [28], Australia [29], U.K. [30], and Holland [31] (Additional file 1). Studies were published between 1996-2010 but more than 50% (n = 17) had been published since 2008. Among studies that reported response rates (n = 20) the lowest was 6.6% [32] and the highest was 64% [33]. Mean ages among samples ranged between 30 and 50 years, although one study that followed participants during and after high school included noticeably younger participants (mean age = 21 years)[34]. Most samples included both men and women except two that included women only [33,35]. All income groups were represented among the studies reviewed.

### Study designs

Thirteen quasi-experiments were reviewed. Among these, physical activity was measured either: 1) in the same respondents before and after relocating to a new neighborhood [33-36]; 2) in the same respondents before and after an environmental modification [37-42], or; 3) in separate respondents before and after an environmental modification [28,30,43]. Two studies attempted to assess temporal precedence by asking respondents to recall their physical activity behaviors before they had relocated to their most recent neighborhood (i.e., quasi-longitudinal)[44,45]. Ten of the quasi-experimental studies included a modification to the built environment (i.e., no residential relocation) of which two included intervention and comparison groups [38,43] and eight included an intervention group only [28,30,33,37,39-42].

Twenty studies used statistical approaches to control for self-selection in the estimated association between the built environment and physical activity. Most of these captured each respondent's reasons for moving to their current neighborhood or their preferences for particular neighborhood attributes and used this information as a covariate in subsequent analysis. The types of preferential attributes included valuing shops and service close by [46], preference for accessibility, physical activity options, or safety [45], or the closeness to school and public transit, desire for nearby shops and services, and ease of walking [29]. Studies included individual [46-48] and multi-item neighborhood self-selection scales [29,32,44,45,49-55]. Only eleven studies provided evidence for the reliability or validity of their neighborhood self-selection items [29,44,45,49-55].

Other statistical approaches were also used to take into account neighborhood self-selection, notably instrumental variables and propensity scoring. Khattack and Rodriguez [56] regressed the respondents neighborhood type (i.e., neo-traditional or conventional) onto attitudes related to residential choice that were not likely associated with travel behavior, and then used the predicted probability from this model when estimating the association between the built environment and walking trips. Greenwald and Boarnet [57] also used instrumental variable analysis, by including aggregate-level variables that were associated with choice of urban setting but not with individual travel behavior (i.e., percent capita income, population with at least a college education, population identified as African American, population identified as Hispanic, population of housing units classified as rural not farms). Cao[53] used propensity score analysis to estimate the conditional probability that an individual resided in a traditional vs. suburban neighborhood based on measured respondent characteristics, including residential preferences, which was then used to adjust the association between neighborhood type and transportation and recreational walking. Boarnet et al.[58] used a Heckman selection model to obtain an estimate of unmeasured characteristics related to neighborhood self-selection, which was then included as a covariate in a model regressing physical activity onto neighborhood type. Pinjari et al. [59] took a slightly different approach by jointly modeling residential choice and the association between the built environment and physical activity.

Studies adjusting for neighborhood self-selection generally used single regression equations, with two (one cross-sectional and one quasi-longitudinal) using structural equation models [44,52].

### Measurement of physical activity

Self-reports were the most common method for capturing physical activity (n = 27 studies), four studies used direct observation [28,30,38,43], three used accelerometers [37,42,55], and one used pedometer data [33] (Additional file 1). Twelve studies measured overall walking, eight captured walking for transport, and seven captured recreational walking. Twelve studies measured moderate-to-vigorous intensity physical activity (MVPA), and six captured cycling behavior. Physical activity undertaken during the last 7 days (n = 11), 30 days or month (n = 5), two days (n = 4) or daily (n = 6) were often captured. Several studies captured context-specific physical activity including physical activity undertaken in the neighborhood [43,45,48,53,54,59], park [38], on a street or cycle path [28,30], and on a trail [40]. Less than half of studies presented supporting evidence for the reliability or validity of their physical activity data collection method [29,33,34,36-42,45,54,55].

### Measurement of the built environment

Application of GIS techniques to existing geographical databases was the most common method for characterizing the built environment. Neighborhoods were generally operationalized as local areas, transportation zones, census districts, or buffers within a 1600, 800 or 400 m Euclidean and network distance of home. The association between land use proximity and mix and density (population and employment) was examined most often among the studies reviewed (Table 1). Natural experiments included installation of trails[40,41,43], cycle lanes [28], transit stops or stations[37,39,42], street lighting[30], and park improvements[38] (Tables 1 and 2).

**Table 1:** Summary of associations between built environmental attributes and physical activity among all studies (cross-sectional and quasi-experiments)

	N total (studies)	Recreation walking	Transportation walking	General walking	General cycling	Combined walk/cycle	Moderate to vigorous PA*
**Neighborhood characteristics**							
**Street/pedestrian connectivity**	5	o[^48^]	+[^48^]	o[^57^]/+[^58^]		+[^47^]	-[^33^]
**Land use mix**	6	o[^45^]	+[^45^]	+[^44^,^45^]	+[^45^]	+[^51^,^60^]	o[^59^]/+[^54^]
**Recreation land use proximity**	7	o[^45^]	o[^45^]	o[^44^]	+[^45^]	o[^60^]/-[^51^]	o[^59^]/+[^34^,^54^]/-[^34^]
**Non-recreational land use proximity**	10	o[^45^,^48^]	+[^45^]/-[^45^,^46^,^48^]	o[^44^]/-[^58^]	o[^45^]	o[^47^,^51^,^60^]	o[^54^,^59^]
**Transit proximity/access**	5			o[^39^]/+[^58^]		+[^47^]	o[^39^,^42^]/+[^37^]
**Population/residential density**	6	+[^35^]/-[^35^]	+[^35^]	+[^57^]		o[^47^]	o[^33^,^34^,^59^]
**Employment/job density**	5			o[^57^]/+[^58^]		o[^47^]	o[^59^]/-[^33^]
**Aesthetics/variety/diversity**	2	o[^48^]	o[^48^]				-[^34^]
**Trails/pathways/cycle ways/sidewalk**	5			o[^31^]/+[^43^] /-[^40^,^41^]	o[^31^,^41^,^47^]/ +[^28^,^43^]/-[^40^]		o[^40^]/+[^43^] /-[^41^]
**Parks/public open space install or improvements**	2			+[^31^]	o[^31^]		o[^38^]
**Pedestrian/cyclist amenities (street furniture, lighting, shading)**	3	o[^48^]	o[^48^]	+[^30^]			o[^59^]
**Traffic-related**	2	o[^48^]	+[^48^]	o[^31^]	o[^31^]		
**Aggregated neighborhood characteristics**							
**Walkability/pedestrian index**	4	o[^29^,^55^]/+[^50^]	+[^29^,^50^,^55^]	+[^57^]		o[^52^]	+[^55^]
**Neighborhood type (traditional, New urbanist)**	5	+[^53^]	+[^53^]	+[^32^,^56^]		+[^49^,^51^]	
**Sprawl**	1			o[^36^]			o[^36^]

**+**: studies reporting statistically significant positive association between the environmental characteristic and physical activity.

**-**: studies reporting statistically significant negative association between the environmental characteristic and physical activity.

**o**: studies reporting no statistically significant association between the environmental characteristic and physical activity.

Cross-sectional results that adjust for residential selection included only. Quasi-experimental studies: [28,30,31,33-43].

* Also included pedometer and accelerometer-determined physical activity and use of specific locations (i.e., parks or trails).

**Table 2:** Summary of temporal associations between built environmental attributes and any physical activity outcome by type of quasi-experimental design

	Change in physical activity behavior		
	**Increase**	**No change**	**Decrease**
	
**Neighborhood built characteristics**			
Street/pedestrian connectivity			
Land use mix			
Recreation land use proximity			
Non-recreational land use proximity			
Transit proximity/access			
Population/residential density			
Employment/job density			
Aesthetics/variety/diversity			
Trails/pathways/cycle ways/sidewalk			
Parks/public open space			
Pedestrian/cyclist amenities			
Traffic-related			
Sprawl			

Association found using a same sample pre-post design (residential relocation)

Association found using a same sample pre-post design (environmental modification)

Association found using a different sample pre-post design (environmental modification)

Association found using a same sample pre-post quasi-longitudinal design

### Associations between the built environment and physical activity

#### Street and pedestrian connectivity

Three studies found positive associations between connectivity and physical activity. Cao et al. [48] found that walking to the store inside the neighborhood during the past month was positively associated with pedestrian connections between the street and stores in a commercial street (i.e., pedestrian entrances). Similarly, Boarnet et al.[58] found that the number of intersections within a census block was positively associated with distance walked while Chattman[47] found a positive association between the number of 4-way intersections and frequency of non-work walking and biking trips. Contrary to these cross-sectional findings, Wells and Yang [33] using a quasi-experiment found women who had moved to a neighborhood with fewer cul-de-sacs walked less than they had before, suggesting a negative association between connectivity and physical activity.

#### Land uses

Six studies found associations between land use mix and physical activity. Handy et al. [45] found that the number of different businesses within 800 m of home was positively associated with the frequency of walking to a store, but not strolling, within the neighborhood. Positive associations between frequency of MVPA[54] and walking and biking[51] and the number of different business types within 400 m of home was also found. In addition, Cao et al. [60] found the monthly frequency of undirected walking and biking trips undertaken during good weather to increase as the number of business types within 1600 m of home increased. Quasi-longitudinal findings from the same dataset suggested that movers reported doing more walking and biking one-year post move if their current neighborhood included an increased mix of businesses [44,45].

Cao et al. [51] found an increase in monthly home-based non-work walking and biking trips with decreased distance to a theatre (attenuated but remained significant after adjustment for residential preferences), and non-significant associations (attenuated and became non-significant after adjustment for residential preferences) between distance to the library and post office and walking/biking trips. Cao et al.[48] found a negative association between distance to the nearest store and monthly frequency of walking to a neighborhood store after adjusting for the respondent's reported importance of having a store within walking distance. Using the same dataset, distance to the nearest grocery store was negatively associated with frequency of walking to stores inside the neighborhood [45]. Boarnet et al.[58] found a negative association between distance to city hall and the distance walked over two days, while residents of a neo-traditional neighborhood were found to do more transportation-related walking trips the closer they resided to a commercial center [46]. Contrary to the expected negative relationship between distance to destinations and physical activity, Handy et al. [45,54] found that increasing distance to the nearest bank and health establishment was associated with more walking. Temporal evidence provided general support for these cross-sectional study findings. Boone-Heinonen et al.[34] found that weekly frequency of MVPA increased among those relocating to a neighborhood with more public and pay recreational facilities, although a decrease in MVPA frequency was found among women relocating to a neighborhood with more pay recreational facilities suggesting sex differences in the built environment-physical activity relationship. Generally this evidence together suggests that increased mix and proximity of land uses are important for encouraging physical activity.

#### Transit proximity and access

Distance walked was found to increase as the distance between home and light rail transit increased [58] while another study found a positive association between the presence of light, but not heavy, rail within 800 m from home and non-work waking or biking frequency [47]. One quasi-experiment found that ridership increased after the installment of a rail stop, which itself was positively associated with accelerometer-assessed moderate-intensity physical activity [37]. Data from the same study however, later showed no group (ridership status)-by-time (pre-post rail installment) nor a time related difference in accelerometer-assessed moderate-intensity physical activity[42]. Moreover, no association between transit use and achieving recommended walking or vigorous physical activity was found after the development of a light rail transit corridor [39]. Overall there appears to be mixed support for the importance of transit (rail) accessibility and physical activity.

#### Population and employment density

Population density was associated with walking behavior in two studies. Greenwald and Boarnet[57] found a positive cross-sectional association between the frequency of non-work related walking and population density, while Coogan et al.[35] based on a quasi-experiment found that women relocating to a neighborhood with a higher population density increased their weekly time spent walking for transportation and recreation. One cross-sectional study showed a significant positive association between employment density and walking distance while also finding that the association between population and retail employment density and walking distance attenuated to non-significance after adjustment for neighborhood self-selection [58]. A quasi-experiment found a negative association between the ratio of service jobs to the number of residents and post-relocation pedometer-determined steps among women [33].

#### Aesthetics and design

Two studies examined the relationship between aesthetics and physical activity. A cross-sectional study found no association between aesthetics-related attributes (i.e., design variation, sidewalk shading, front door setback, and proportion of houses with porches) and monthly frequency of neighborhood strolling and transportation-related walking [48]. A quasi-experiment found a counterintuitive reduction in the weekly frequency of MVPA among respondents after relocating to a neighborhood with more landscape diversity [34]. Based on the few studies reviewed there appears to be limited evidence for aesthetics supporting physical activity.

#### Pedestrian and cyclist infrastructure (including sidewalks, trails and pathways)

Chattman [47] found a positive association between sidewalks on both sides of the street and the number of non-work trips by walking/biking, however this was no longer statistically significant after adjusting for neighborhood self-selection. The installment of an urban greenway/trail, was associated with increases in walking, cycling and other physical activity post-intervention [43]. These results were supported by another study which found increases in daily bicycle traffic following the installation of cyclist infrastructure (i.e., cycle lanes, signage, marking, reductions in speed limits) [28]. In addition, Painter[30] found an increase in the number of pedestrians using the street following installation of street lighting in three neighborhoods. Several studies however, have show no association between pedestrian or cyclist infrastructure and physical activity. For instance, a quasi-experiment by Meurs and Haaijer [31] found no change in weekly walking and bicycle trips among people relocating to neighborhoods with more or less supportive street characteristics such as access to cycle paths, pedestrian priority areas, and traffic calming strategies. Furthermore, installation of a new trail was not found to increase physical activity, and in fact was associated with a reduction in the likelihood of time spent walking and cycling among trail users[40]. This finding might suggest that the increased connectivity resulting from installation of the new trail meant that people using the trail as transport corridor do need to spend as much time walking or cycling to reach destinations. Goulias and Burnbridge[41] found counterintuitive reductions in walking and overall physical activity frequency 5-months after the construction a neighborhood multi-use trail [40,41]. Whether or not pedestrian and cyclist infrastructure lead to increases in physical activity behavior might depend on the type of attributes and the extent of the environmental modification undertaken and the type of physical activity behavior evaluated.

#### Neighborhood parks and open space

Meurs and Haaijer[31] examined changes in the neighborhood environment among movers and non-movers during a nine year period and found that increases in the quantity of parks, green strips, and playgrounds to built environment positively associated with weekly walking, but not cycling, trips among non-movers. Although the same relationship was not found among movers. Cohen et al. [38] studied the effect of neighborhood park upgrades on park use and physical activity. They found that park use and exercise did not change as a result of the upgrades (i.e., new or redesigned gymnasium, field improvements, landscaping, walking paths, picnic areas, playgrounds) although the number of first time parker users did increase.

#### Traffic-related characteristics

Cao et al. [48] found a cross-sectional association between reductions in traffic volume and increases in monthly frequency of walking to the store, but not strolling, in the neighborhood however, no association was found in another study examining changes in weekly walking and cycling trips after participants moved to a street with traffic calming and 30 km/h zones [31].

#### Walkability, sprawl, and neighborhood type

Four studies examined the association between a neighborhood walkability or pedestrian index and physical activity. Positive cross-sectional associations were found between neighborhood walkability - combination of commercial floor space, land use mix, residential density, and connectivity - and walking trips[50]. In addition, those who preferred less walkable neighborhoods were generally less likely to walk for any purpose regardless of whether they lived in a high or low walkable neighborhood [50]. Sallis et al. [55] and Owen et al. [29] using a walkability index with the same components (intersection density, retail density, retail floor area, and land use mix) found positive associations with transportation-related, but not recreational, walking. Specifically, Sallis et al. [55] found that minutes of leisure walking in high walkable neighborhoods attenuated after adjusting for reasons for moving to the neighborhood. They also found more accelerometer-determined MVPA in high versus low walkable neighborhoods. Owen et al. [29] found that neighborhood walkability was associated with more frequent transportation walking among respondents who reported desire for nearby shops and services as important reasons for moving to their current neighborhood. These findings are supported by Greenwald and Boarnet[57] who found a positive cross-sectional association between a neighborhood pedestrian environment score -- a composite score based on ease of street crossing, sidewalk continuity, street connectivity, and topography -- and frequency of non-work walking trips. In contrast Bagley and Mokhtarian[52] found no association between suburban and traditional neighborhood indices and daily walking/biking distance.

Neighborhood type was consistently associated with physical activity behavior including transportation-related, recreational, non-specific walking and cycling[32,49,51,53,56]. The findings of these studies together albeit cross-sectional, suggest that neighborhoods with higher residential densities, more mixed land use, and that include highly connected street patterns are more supportive of pedestrian and cyclist activity than neighborhoods that do not have these characteristics. One study only examined changes in physical activity in relation to composite environmental sprawl index which included measures population density, percent of people residing in high or low densities areas, population density per square mile of urban land, mean block size, and percent of blocks ≤ 500 feet on a side [36]. The study showed no association between change in physical activity energy expenditure nor walking distance among movers to a different county regardless the level of sprawl. Nevertheless, the combination of different environmental attributes appears to important for supporting physical activity.

## Discussion

To date reviews of studies examining the association between the built environment and physical activity have not summarized findings based on study control of neighborhood self-selection [8,10,24,25]. This review extends prior knowledge by summarizing findings from studies that attempt to address neighborhood self-selection and temporal precedence when examining the association between the objectively-assessed built environment and physical activity. We found that the associations between specific built environmental attributes and physical activity were generally mixed based on this more rigorous evidence. Land use mix, composite walkability indices and neighborhood type were nevertheless consistently associated with higher physical activity levels even after controlling for neighborhood self-selection. Moreover, the built environment was found to be more supportive of walking and cycling compared with physical activity more generally-congruent with previous findings [10,24,25] and with current knowledge regarding behavior-specific environmental settings [61].

Our finding that associations between the built environment and physical activity exist from both cross-sectional studies that adjust for neighborhood self-selection and from quasi-experiments is promising. To date few cross-sectional studies have adjusted for neighborhood self-selection when examining the relation between the built environment and physical activity. This is reflected in the low number of included cross-sectional studies in this review versus elsewhere [8,10,24,25]. Cross-sectional findings that do not account for residential self-selection provide no indication of the extent to which changes in the built environment might be independently associated with changes in physical activity. They may instead just reflect an active individual's preference to live in neighbourhoods that support their existing physical activity levels. In practice, the built environment likely has some effect on the amount of physical activity engaged in by active people who desire to reside in more walkable neighborhoods[50] and the balance between neighborhood supply and demand has the potential to influence population-levels of physical activity.

The purpose of this study was not to describe how neighborhood self-selection and its potential interaction with the built environment influences physical activity but rather to isolate the influence that changes in the built environment might have on changes in physical activity. A unique finding of our review is that the association between the built environment and physical activity likely exists independent of residential location choices. The importance of adjusting for neighborhood self-selection in cross-sectional studies is indicated by reviewed studies that showed an attenuation in the association between the built environment and physical activity after adjustment [47,51,55]. Future cross-sectional studies should therefore try to account statistically for neighborhood self-selection in order to obtain more robust estimates of the likely magnitude of any associations between changes in the built environment and physical activity.

Some support for the idea that changes in the built environment precede changes in physical activity is found in the quasi-experimental studies reviewed here that show increased cyclist activity after the installment of cycling infrastructure[28], increased walking, cycling, and other physical activity after installment of a greenway trail [43], increased pedestrian activity following installment of street lighting[30] and increased first time park user following park upgrades [38] (Table 2). These results suggest that modifying one or a few environmental attributes independent of other factors has the potential to encourage more physical activity. However, null and counter-intuitive associations were also found, for example, the reduction in physical activity following the installation of a trail [40,41]. The overall pattern of findings from quasi-experimental evidence also suggests an increased number of null associations from studies with stronger designs (Table 2). Quasi-experiments offer more robust evidence of causality compared with cross-sectional designs however, they are still subject to biases that might contribute to counter-intuitive findings[27]. The use of quasi-experimental studies in the field is in its infancy. Future quasi-experiments of built environment interventions should take steps to rule out competing explanations and biases such as capturing pre and post intervention data from cohorts, taking multiple pre and post intervention measurements, including multiple intervention-matched control groups and obtaining measures of individual-level dose or exposure to the intervention [27]. Capturing information about intermediate variables (e.g., psychosocial factors, knowledge, awareness) in addition to physical activity might also provide a better understanding about the pathways by which the built environment influences physical activity or clues when interventions provide unexpected results.

To date few potential built environmental interventions and their influence on physical activity have been evaluated and published in the peer-reviewed literature. In addition, environmental attributes that are modified are often those that are only modestly or inconsistently associated with physical activity [8,10,24,25]. There is a dearth of studies examining changes in physical activity among the same respondents in the same neighborhood following changes in pedestrian connectivity, population density, or land uses - which are consistent correlates of walking. This suggests quasi-experiments examining changes in behavior pre and post installment or modification of a wider range of environmental features is needed. For this to occur researchers need to know when changes to the built environment are scheduled to take place to design the study methodology and to obtain resources. Changes to funding opportunities (i.e., rapid reviews for natural experiment opportunities) and building stronger partnerships with urban designers, city planners, and community groups might alleviate some of the barriers to conducting quasi-experiments.

Despite providing preliminary evidence for a causal association between the built environment and physical activity several limitations should be considered when interpreting the findings of our review. The inclusion of only peer-reviewed studies added to the scientific rigour of our review however this approach also means that our findings likely are affected by publication bias thus overestimating the potential influence of environmental attributes on physical activity. The focus on only peer-reviewed evidence might have resulted in the inclusion of fewer quasi-experiments as many urban planning departments likely do not publish evaluations of their own interventions within this forum. We did not exclude or weight study findings based on their individual rigor. This is important to note given that many studies did not provide estimates of the reliability of their physical activity or residential self-selection items. Given the small number of studies included in this review we did not differentiate among quasi-experimental approaches nor method of neighborhood self-selection adjustment. A discussion of the different methods of accounting statistically for neighborhood self-selection, including strengths and limitations is presented elsewhere [23]. We grouped environmental attributes in categories that have face validity, but we acknowledge that some attributes could be located under multiple categories that may not be mutually exclusive.

Data for some environment-physical activity associations were not available from the studies reviewed (e.g., aesthetics and cycling) or if available were examined in only a few studies. Between studies, there was also wide variation in the methods used to measure physical activity and the built environment. Therefore making definitive statements about which environmental attributes should be targeted to increase specific types of physical activities is difficult from our findings. Nevertheless, in support of findings elsewhere [10,24,25], our results suggest that some specific environmental attributes might be associated with certain physical activities. For example, connectivity, land use mix, and traffic-related factors are associated with walking for transport but not recreational walking, and population density is associated with walking but not cycling for any purpose or non-specific moderate-to-vigorous intensity physical activity. Consistency in the measurement of built environment attributes and physical activity across studies would improve the comparability of findings and result in stronger statements about the relationship between the built environment and physical activity. This might also allow quantitative synthesis techniques such as meta-analysis to be used.

Evidence regarding the association between the built environment and physical activity found from this review is mixed. Generally, the associations between attributes of the built environment and physical activity from cross-sectional studies appear to remain following statistical adjustment of self-selection although associations attenuate somewhat. However, more rigorous quasi-experimental studies of the relationship between the built environment and physical activity provide less support, with several positive, null, and even counter-intuitive negative associations being found. The mixed nature of findings from the few quasi-experimental studies that have been undertaken on this topic to date, suggest that more quasi-experimental research is needed in order to provide stronger evidence for recommending the creation of walkable neighborhoods as an effective population health intervention for increasing physical activity.

Creating or modifying neighborhoods to make them walkable may not always immediately lead to more physical activity, but could result in other health benefits such as higher social capital [62,63], improved mental health [64], and fewer motor vehicle caused pedestrian injuries [65]. However, there is dearth of data on the economic cost of improving neighborhood walkability (or health supportiveness) and the potential subsequent health care savings resulting from improvements in health. Future studies should examine the potential short and long-term effects of built environment interventions on behavior as well the cost-effectiveness of creating walkable neighborhoods. The results of this review reflect previous evidence regarding the importance of street and pedestrian connectivity, land use and destination mix, population density, and overall neighborhood design for supporting physical activity among adults, and adds to the evidence by showing that these associations tend to remain even after taking steps to control for neighborhood self-selection.

## Competing interests

The authors declare that they have no competing interests.

## Authors' contributions

GRM and AS conceived the study, conducted the synthesis, interpreted the findings and contributed to writing of the manuscript. All authors read and approved the final manuscript.

## Supplementary Material

Additional file 1 **Summary of extracted information from studies included in the review (n = 33)**. The file includes information about the study design, sample recruitment, sample characteristics, physical activity variables, built environment variables, neighborhood self-selection, confounders, and findings for each study included in the review. Click here for file
