# Supplementary material for: In search of causality: a systematic review of the relationship between the built environment and physical activity among adults
Source: Int J Behav Nutr Phys Act. 2011 Nov 13;8:125. doi: 10.1186/1479-5868-8-125 (PMC3306205; doi:10.1186/1479-5868-8-125)
Supplement: Additional file 1 — Summary of extracted information from studies included in the review (n = 33). The file includes information about the study design, sample recruitment, sample characteristics, physical activity variables, built environment variables, neighborhood self-selection, confounders, and findings for each study included in the review. [file 1479-5868-8-125-S1.DOC]

**Additional file 1: Summary of extracted information from studies included in the review** (n=33)

| **First author** | **Study design** | **Sample recruitment** | **Sample characteristics** | **Physical activity variable** | **Built environment variables** | **Residential selection adjustment** | **Confounding variables** | **Findings** |
| --- | --- | --- | --- | --- | --- | --- | --- | --- |
| Frank[50] | Cross-section, 13 Counties, Atlanta, US, spring & fall 2001 & 2002 | Drawn from households selected based on income, size, & residential density (R=30.4%) | Neighborhood selection sample (n=2088):  36.9±12.2yrs of age  52.8% women  $40-49999 annual household income  ≤3yrs in current neighborhood  Neighborhood preference sample (n=8069):  51.1±13.8yrs of age  53.3% women  $50-59999 annual household income | Self-reported non-discretionary & discretionary walking trips from a 2-day travel diary | Walkability index. Sum of commercial floor index, land-use mix, net residential density, connectivity w/in a 1-km road network buffer | Statistical adjustment using measured self-reported indicators of residential preference/reasons for moving to neighborhood | Socio-demographics | 3^rd^ & 4^th^ walkability quartiles associated with “any walking”(+) & “any non-discretionary walking”(+) in neighborhood selection sample only  4^th^ walkability quartile associated with “any discretionary walking trips”(+) in neighborhood selection sample only  Preference of low walkable neighborhood associated with low walking of any kind regardless of walkability of neighborhood. |
| Bagley[52] | Cross-section, 5 neighborhoods, San Francisco Bay Area US, 1992 | Respondents randomly selected from neighborhoods selected based on variation in land use type, transit accessibility, land uses, residential density, employment mix  (n=963, R=18%, analytic n=515) | 44.8±9.4yrs of age  55% women  $35-50000 annual income  24.7±15.6yrs residing in Bay Area | Self-reported daily walk/bike distance traveled | Two indices: 1) traditional neighborhood index 2) suburban neighborhood index based on parking availability, distance to nearest transit & grocery store, presence of sidewalks, mean speed limit, street pattern, population density, perceived pleasantness of walking/cycling in neighborhood | Statistical adjustment (structural equation model) using measured self-reported indicators of residential preference/reasons for moving to neighborhood | Socio-demographics, lifestyle, attitudinal factors | Residential location not associated with daily walk/bike travel distance |
| Cao[48] | Cross-section, 6 neighborhoods (selected based on era: traditional, early modern, late modern), Austin, Texas, US, May 1995 | Respondents recruited from randomly selected households (n=1368; R=23%) | 42-44.3yrs (median age)  50-58% men  $42-69889 annual household income (median)  6.2-12.4yrs residing in the current home (mean) | Self-reported frequency 1) strolling & 2) walking to the store in the last 30 days in the neighborhood | Land area, street miles, street mile/land area, street area, street area/land area, % T-intersections, intersections/street mile, cul-de-sacs/street mile, blocks/land area, access points/land area, % HH w/in walk distance of commercial street, location of shop entrances, traffic volume (commercial street), share of road w/sidewalk, level of sidewalk shading, street width, front door setback, % house w/porches, design variation | Statistical adjustment using measured self-reported indicators of residential preference/reasons for moving to neighborhood | Socio-demographics, neighborhood perceptions, local commercial area characteristics | Strolling frequency not associated with built environment adjusting for importance of stores w/in walk distance  Walking to store frequency associated with traffic volume (-) & pedestrian connection (+) in the commercial street, & distance to nearest store (-) adjusting for importance of stores w/in walk distance |
| Cao[60] | Cross-section, 8 neighborhoods (traditional & suburban selected based on size of metropolitan area & region), Northern California, US, late 2003 | Respondents recruited from randomly selected households (n=1682; R=25%) | 46.9-58.2% women  43.3-54.7yrs (mean age)  $45500-98700 annual household income (median) | Self-reported frequency of undirected walking/biking trips in a typical month with good weather (6-point scale from never to ≥2 times/wk) | # different business establishments w/in 1600m, distance to nearest establishment of each type, # establishments of each type w/in 1600m, based on street network from respondents home  Business establishments included institutional (bank, church, library, post office), maintenance (grocery store, pharmacy), eating-out (bakery, pizza, ice-cream, & take-out), leisure (health club, bookstore, bar theatre, & video rental) | Statistical adjustment using measured self-reported indicators of residential preference/reasons for moving to neighborhood | Socio-demographics, travel attitudes, neighborhood perceptions | Frequency of walking/biking associated with # of business types w/in 1600m (+) adjusting for preference for neighborhood physical activity options |
| Cao[51] | Cross-section, 8 neighborhoods (traditional & suburban selected based on size of metropolitan area & region), Northern California, US, late 2003 | Respondents (movers in previous year & non-movers) recruited from randomly selected households neighborhoods (n=1682; R=25%) | 46.9-58.2% women  43.3-54.7yrs (mean age)  $45500-98700 annual household income (median) | Self-reported frequency of home-based non-work walking/ biking trips in a typical month with good weather (6-point scale from never to ≥2 times/wk) | Neighborhood type (traditional vs. suburban), # business type w/400m, # business type w/800m, # institutional business w/800m, distance to nearest library, distance to nearest theatre, distance to nearest post office) based on street network from respondents home  Business establishments included institutional (bank, church, library, post office), maintenance (grocery store, pharmacy), eating-out (bakery, pizza, ice-cream, & take-out), leisure (health club, bookstore, bar theatre, & video rental) | Statistical adjustment using measured self-reported indicators of residential preference/reasons for moving to neighborhood | Socio-demographics, travel attitudes, neighborhood perceptions | Walking/biking frequency higher in traditional vs. suburban neighborhood type adjusting for residential preferences  Walking/biking frequency associated with # business types w/400m (+) adjusting for residential preferences  Walking/biking frequency associated with distance to nearest theatre (-) adjusting for residential preferences  Walking/biking frequency association with distance to nearest library and post office attenuated to non-significance after adjustment for residential preferences |
| Cao[53] | Cross-section , 8 neighborhoods (traditional & suburban selected based on size of metropolitan area & region), Northern California, US, late 2003 | Respondents recruited from randomly selected households (n=1682; R=25%)  (analytic sample>=1542) | 46.9-58.2% women  43.3-54.7yrs (mean age)  $45500-98700 annual household income (median) | Self reported frequency 1) strolling & 2) walking to the store in the last 30 days in neighborhood | Suburban vs. traditional neighborhood type  Traditional neighborhoods (# business w/in 400m=2.6; w/in 1600m=13.0; minimum distance to: any business=247m; institution=377m; maintenance=380m; eat-out=526m; leisure=508m)  Suburban neighborhoods  (# business w/in 400m=0.8; w/in 1600m=9.6; minimum distance to: any business=557m; institution=760m; maintenance=819m; eat-out=789m; leisure=814m) | Statistical adjustment using measured self-reported indicators of residential preference/reasons for moving to neighborhood | Socio-demographics, travel attitudes | Frequency of utitilitarian walking & strolling higher in traditional vs. suburban neighborhood adjusting for residential preferences (attractiveness, spaciousness, accessibility, safety, physical activity options, socializing) via propensity score adjustment |
| Cao[44] | Quasi-longitudinal, 8 neighborhoods (traditional & suburban selected based on size of metropolitan area & region), Northern California, US, late 2003 | Respondents (movers in previous year & non-movers) recruited from randomly selected households neighborhoods (n=1682; R=25%)  (analytic sample=547 movers) | 48-74% women  35.6-49.4yrs (mean age)  $50000-$110000 annual household income (median) | Self-reported change in current walking behavior compared with one year ago (captured on a 5-point scale from “a lot less now” to “a lot more now) | # different business establishments w/in specified distances, distance to nearest establishment of each type, # establishments of each type w/in specified distances, based on street network from respondents home  Business establishments included institutional (bank, church, library, post office), maintenance (grocery store, pharmacy), eating-out (bakery, pizza, ice-cream, & take-out), leisure (health club, bookstore, bar theatre, & video rental) | Statistical adjustment using measured self-reported indicators of residential preference/reasons for moving to neighborhood | Socio-demographics, travel attitudes, neighborhood perceptions, driving behavior | Relative change in walking associated with # business types w/in 400m in the current neighborhood (+) adjusting for changes in preference of neighborhood attractiveness, spaciousness, accessibility, safety, physical activity options |
| Handy[54] | Cross-section, 8 neighborhoods (traditional & suburban selected based on size of metropolitan area & region), Northern California, US, late 2003 | Respondents recruited from randomly selected households in (n=1682; R=25%; analytic n=1497) | 46.9-58.2% women  43.3-54.7yrs (mean age)  $45500-98700 annual household income (median) | Self-reported frequency of moderate-vigorous intensity (MVPA) physical activity (≥10min) in neighborhood in last 7 days | # different business establishments w/in specified distances, distance to nearest establishment of each type, # establishments of each type w/in specified distances, based on street network from respondents home  Business establishments included institutional (bank, church, library, post office), maintenance (grocery store, pharmacy), eating-out (bakery, pizza, ice-cream, & take-out), leisure (health club, bookstore, bar theatre, & video rental) | Statistical adjustment using measured self-reported indicators of residential preference/reasons for moving to neighborhood | Socio-demographics, travel attitudes, neighborhood perceptions | MVPA frequency associated with # business types w/400m (+) & distance to nearest health club (+) adjusting for change in residential preferences (physical activity options, socializing, attractiveness, safety) |
| Handy[45] | Cross-section/quasi-longitudinal, 8 neighborhoods (traditional & suburban selected based on size of metropolitan area & region), Northern California, US, late 2003 | Respondents (movers in previous year & non-movers) recruited from randomly selected households in (n=1682 with valid address; R=25%; analytic n=1328-1534) | 46.9-58.2% women  43.3-54.7yrs (mean age)  $45500-98700 annual household income (median) | Self reported frequency 1) strolling & 2) walking to the store w/in the neighborhood in the last 30 days in neighborhood  Frequency of walking to selected destinations in a typical month with good weather  1 yr change in 1) walking & 2) biking among movers & non-movers measured on a 5-point ordinal scale (a “lot less” to a “lot more” now). | # different business establishments w/in 400, 800, 1600m, distance to nearest establishment of each type, # establishments of each type w/in 400, 800, 1600m, based on street network from respondents home  Business establishments included institutional (bank, church, library, post office), maintenance (grocery store, pharmacy), eating-out (bakery, pizza, ice-cream, & take-out), leisure (health club, bookstore, bar theatre, & video rental) | Statistical adjustment using measured self-reported indicators of residential preference/reasons for moving to neighborhood | Socio-demographics, travel attitudes, neighborhood perceptions | Walking to store frequency associated with # business types w/in 800m (+) & distance to nearest grocery store (-) adjusting for residential preferences (physical activity options, safety, stores w/in walking distance, cul-de-sacs)  No association between the built environment strolling frequency  Relative change in walking associated with distance to nearest bank (+), # banks w/in 800m (+), & # business type w/in 1600m (+)but residential preferences not significant & not included in model  Relative change in biking associated with # maintenance businesses w/in 1600m (+) & distance to nearest health establishment (+) adjusting for residential preferences (spaciousness, attractiveness) |
| Chattman[47] | Cross-section, San Diego & San Francisco-Oakland-San Jose, California, US, November 2003-April 2004 | Stratified random sample of households with over-sampling of households near 12 selected rail stations (n=1113; RR=20%; analytic n=999) | 46.2±17.0yrs of age  57% women  $56900±37900 annual household income  67-72% moved home in previous 10yrs (census statistics for study areas) | Self-reported  frequency of non-work walk/bike trips in previous 24 hrs | # retail workers w/in 400m & 1.6km, #residents/road mile w/in 1.6km, # 4-way intersections w/in 400m, presence of heavy rail station w/in 800m, presence of light rail w/in 800m, distance to nearest major business district | Statistical adjustment using measured self-reported indicators of residential preference/reasons for moving to neighborhood | Socio-demographics, survey areas, neighborhood perceptions | Frequency of walk/bike trips associated with # 4-way intersections (+), light rail access w/in 800m (+) adjusting for searching for neighborhood a with walk &/transit access, & auto access. The association between sidewalks on both sides of the street (+) and walk/bike trips attenuated to non-significance after adjustment for neighborhood search criteria |
| Sallis[55] | Cross-section, 32 neighborhoods (stratified by low/high income & walkability)  King-County WA & Baltimore-Washington DC, US, 2002-2005 | Randomly sampled participants (n=2199, R=26% of which 87% retained in 6mth follow-up survey | 45.1±11.0yrs of age  48.2% women | Accelerometer data converted to moderate-to-vigorous-intensity physical activity (MVPA) min/day using established cut-points  Self-reported minutes 1) transport walking & 2) leisure walking in the last week | Walkability index. Sum of the normalized z-scores for the: intersection density (weight by 2), net retail density, retail floor area ratio, land use mix. | Statistical adjustment using measured self-reported indicators of residential preference/reasons for moving to neighborhood | Socio-demographics | Minutes of MVPA & transport walking higher in high vs. low walkable neighborhoods adjusting for reasons for moving to current neighborhood (desire for nearby shops/services, ease of walking, closeness to recreational facilities)  Minutes of leisure walking not associated with walkability (attenuated) after adjusting for reasons for moving to current neighborhood (desire for nearby shops/services, ease of walking, closeness to recreational facilities) |
| Owen[29] | Cross-section, 32 neighborhoods (clusters: 3-9 adjacent census districts stratified by low/high income & walkability), Adelaide, Australia, July 2003-June 2004 | Randomly sampled participants from households w/in neighborhoods (n=2650; R=11.5%) | 50.8% 45-65yrs of age  63.6% women  40.7% $32-77999 annual household income | Self-reported frequency & minutes 1) transport walking & 2) leisure walking in the last week | Walkability index Sum of intersection density, net retail density, retail floor area ratio, land use mix deciles (index 4-40 higher=more walkable) | Statistical adjustment using measured self-reported indicators of residential preference/reasons for moving to neighborhood | Socio-demographics | Frequency of transport, but not leisure, walking associated with walkability index (+) adjusting for reasons for moving to current neighborhood (closeness to job/school, closeness to public transport, desire for nearby shops/services, ease of walking)No attenuation.  Walkability index associated with more frequent walking if residents reported desire for nearby shops/service as important (moderation) |
| Joh[32] | Cross-section, 16 areas (9 mixed-use centers/7 auto-oriented corridors)  South Bay Region, Los Angeles, US, 2005-2007 | Participants recruited from households w/in neighborhoods (n=2399, R=6.62%; analytical n=1564) | 51.1% women  56.6% ≥$70000 annual household income | Self-reported count of walking trips from a 1-day travel diary | The 15 areas (8 mixed-use/7 auto-oriented) were used as area/neighborhood level indicators of the built environment (1 mixed-use area dropped from the analysis) | Statistical adjustment using measured self-reported indicators of residential preference/reasons for moving to neighborhood | Socio-demographics | Total walking trips higher in 5/8 mixed-use areas vs. 1/4 areas auto-oriented areas adjusting for the importance of neighborhood attributes (able to walk to nearby stores/restaurants, to walk to work, <10min drive to work, good schools, nearby entertainment, transport, vibrant street life, friendly people). No evidence of attenuation in coefficients following adjustment for importance of attributes in the neighborhood. |
| Greenwald[57] | Cross-section,  Greater Portland Area, Oregon, US, 1994 | Not stated | Not stated | Self-reported frequency of non-work walking trips from 2-day diary | % of area in ¼ mile buffer covered by grid, population density/sq. mile, density of retail employment 1 mile from home, Pedestrian Environment Factor (composite score generated from: ease of street crossing, sidewalk continuity, street connectivity, & topography) | Statistical adjustment using instrumental variables model to represent choice of residential location. | Socio-demographics, trip cost | Frequency of non-work walking associated with population density (+) pedestrian environment score (+) adjusting for instruments capturing choice of residential location (per captia income in area, % population in area with at least college education, % population in area identified as Hispanic, % housing units in area classified as rural/not farms, % housing units in area classified as urban dwellings. No evidence of attenuation in coefficients following adjustment for choice of residential location. |
| Pinjari[59] | Cross-section, geographical stratified counties & districts, San Francisco Bay Area, California, US, 2000 | 1 respondent/  randomly selected household (n=15066; R=15.6%; analytical n=2793) | Respondents from 1 Bay Area county (Almeida County)  ≥16yrs of age | Self-reported non-work time/day allocated to out-of-home pure physically active recreation (walking, jogging, bicycling around neighborhood), & out-of-home physically active recreation (playing tennis, exercising at gym etc.) from a 2-day travel diary (one randomly chosen weekday) | For each traffic analysis zone: size/density (population, employment, household), land-use structure (housing type, % residential/ commercial, land use mix), regional accessibility (shopping, recreation, employment), activity opportunity (# business establishments/  mile^2^ (recreation, maintenance, physically active, physically inactive recreation, eat-out businesses), transport networks (highway, bikeway, local road densities) | Statistical adjustment using a joint residential choice time-use model. | Socio-demographics, travel behavior, day of week, season | Association between out-of-home physically active pure recreation bicycling facilities (+) not significant after adjustment for observed & unobserved residential choice factors including (zone environmental attributes, socio-demographics, employment, household-level commute variables). |
| Brown[37] | Quasi-experiment (single-group pre/post-test design) Salt Lake City neighborhood, Utah, US, summer 2005/2006 | Respondents residing within ½ mile of new rail stop recruited via door-to-door visits (n=215; R=47.44%; analytic n=47 with follow-up accelerometer data) | 47% women  41±13.83yrs of age  $24000-43367 annual household income (neighborhood mean)  5±7yrs residing in neighborhood | Accelerometer counted bouts/hr of moderate-intensity activity | A new rail stop added between two existing stops. New stop added to a neighborhood with industrial land uses, subsidized apartment, multifamily, & small single-family detached housing, grid street pattern, tree-lined sidewalks, with few walkable destinations beyond convenience stores | Accounted for in the study design: same respondents from same neighborhood measured pre & post intervention. | Socio-demographics, activity (pre-test accelerometer data) | Rail ridership increased after installation of new rail stop. Post-test moderate activity bouts was associated with post intervention rail rides (+) adjusting for baseline moderate activity bouts |
| Cohen[38] | Quasi-experiment (multi-group pre/post-test design), 10 urban parks (5 intervention /5 control matched on size, amenities SES). Southern California, US, December 2003-March 2008 | Park users: systematically recruited from the most/least busy park areas (sample pre n=768/post n=712)  Residents recruited from households w/in 0- ¼, ¼-½, ½- 1 mile, 1-2 miles of each park (pre n=767/post n=620) | Park users at pre/post, respectively:  36.5/37.1yrs (median age)  46.2/37.5% men  54.8/44.2% resided in neighborhood ≥5yrs  Residents at pre/post, respectively:  38.5/40.5yrs (median age)  37.8/28.1% men  58.2/47.5% resided in neighborhood ≥5yrs | Self-reported frequency 1) park use/week & 2) exercise 3 times/wk during leisure time & % first-time park users  Direct observation of park use at 4 time points/day for 7 days | Intervention park upgrades: new gymnasiums (in 3 parks), redesign gymnasium (1 park), field improvements in watering & landscaping (1 park), improvements to picnic areas, walking paths, & playground (1 park) | Accounted for in the study design: same households measured pre & post intervention | Socio-demographics, environmental characteristics, activity | Park use & exercise not associated with park improvement but % first-time users was higher in improvement parks pre to post intervention vs. control parks |
| MacDonald [39] | Quasi-experiment (single-group pre/post-test design), Charlotte, North Carolina, US, July 2006-July 2008 | Residents from households w/in census tracts w/in 1 mile radius of the new Light Rail Transit (LRT) corridor (pre n=839; R=45%/post n=489) | LRT users:  46.2% men  42.3yrs (mean age)  LRT non-users:  46.2% men  46.6yrs (mean age) | Self-reported total days & usual time/wk of 1) walking & 2) vigorous activity measured at baseline & 6-8 months post intervention | Intervention included a new LRT corridor. Built environment variables captured (residential density, park w/in ½ mile radius of home, density of food & alcohol establishments w/in ½ mile radius of home) were used in baseline cross-sectional comparisons & not presented here | Accounted for by study design: same respondents measured pre & post intervention | Socio-demographics, public transit use, neighborhood perceptions, social environment, intentions | LRT use to commute to work not associated with increases in walking or vigorous physical activity recommended levels following the construction of the LRT |
| Shay[46] | Cross-section, one neo-traditional neighborhood, Southern Village in Chapel Hill, North Carolina, US, Spring 2003 | n=348 (no other information available) | 40.8% men  41.3±14.0yrs of age | Self-reported  Neighborhood transport walking trips captured from 24 hour diary (included 99% weekday recalls) | Ariel (linear) distance from home to commercial/retail center (clustered at neighborhood core) | Statistical adjustment using measured self-reported indicators of residential preference/reasons for moving to neighborhood | Socio-demographics, attitudes | # transport walking trips associated with distance to commercial/retail center  (-) adjusting for residential preferences (values shops/services close by) |
| Wells[33] | Quasi-experiment (single-group pre/post-test design), Georgia, Alabama, Florida, US, 2003-2006 | Sample selected from women who had partnered with the self-help housing organization (Habitat for Humanity) & who were moving to a new neighborhood (longitudinal sample n=32; R=64%) | Pre/post sample:  38yrs (mean age)  $16452 annual household income (mean) | Pedometer steps collected pre/post move. Post move steps/wk used as outcome | Neighborhood street network (total linear length of streets, # street intersections, # cul-de-sacs within ¼ mile of home via the network), density (population, employment, housing, & service-job measured at the census tract level or Transit Analysis Zone), land use mix (service-job/population ratio, job/resident ratio) measured at census tract level/Transit Analysis Zone) | Accounted for by study design: same respondents measured pre & post move. Built environment measured in the old & new neighborhoods | Socio-demographics, activity (pre-move pedometer steps). | Post-move pedometer steps associated with moving to neighborhood with more cul-de-sacs (-) & more services jobs/residents (-) |
| Painter[30] | Quasi-experiment (single-group pre/post-test design), Streets in North London, East London, West London, UK, spring | On-street participant recruitment | Not presented | Direct observation of pedestrian count using the 3 streets before (6 wks) & after (6 wks) intervention. between 17:00-23:30 for 10 evenings | Intervention included installation of street lighting in 3 streets in 3 different areas of London 1 week after pre-testing. Installed lighting schemes met British Standards corresponding to high crime areas (including white light lamps). Lighting Changes in night-time environment were significant.  North London (two thoroughfares including street & pedestrian footpath, including high-density multi-story housing, commercial & shops), East London (properties partly boarded, including high-density multi-story housing, hostels, public housing), West London (badly lit, private/ rented accommodation, commercial & shops) | Accounted for by study design: although not necessarily the same participants measured pre & post-intervention in the same 3 streets. | Sex-stratified | Pedestrians counts increased in all sites after lighting improvements |
| Schwanen[49] | Cross-section, 3 neighborhoods (North San Francisco/ NSF, Pleasant Hill/PH, Concord/CON), San Francisco Bay Area, US, May 1998 | Respondents n=2000; R=25% recruited but only subset n=1358 workers commuting once/mth (analytic n=1303) | Not presented | Self-reported walking/jogging/bicycling miles for short-distances (<100 miles one way) during typical 7-day/wk. Use of mode (yes/no) also included as an outcome | The 3 neighborhoods which differed in their environments were compared with each other  NSF (high density, businesses throughout, grid pattern, wide sidewalks, 21 bus routes, no BART access, hilly), PH (medium density, businesses near BART & freeway, fragmented street pattern, BART access, 3 bus routes, discontinuous sidewalks, no hills), CON (low density, businesses at west end, radiating street pattern, BART access, 3 bus routes, discontinuous/  missing sidewalks, no hills) | Statistical adjustment using measured self-reported indicators of residential preference/reasons for moving to neighborhood  (mismatched respondents based on preferences & neighborhood lived in) | Socio-demographics, personality/lifestyle, travel attitudes | Walking/jogging/  bicycling participation more likely among NSF respondents when adjusting for neighborhood type dissonance  Walking/jogging/  bicycling distance similar among the 3 neighborhood types, adjusting for neighborhood type dissonance |
| Meurs[31] | Quasi-experiment (multiple-group pre/post-test design), Holland, 1990-1999 | Respondents included those who had participated in the Dutch Time Use Study in 1990 & who participated in a survey in 1999 (n=189 movers; n=524 non-movers | Not presented. | Self-reported walking & bicycling trips in week from a 7-day diary | Observed street (direct access to cycling path, easy parking, pedestrian priority area, traffic calming, 30km/h zone ) & neighborhood (parks/green strips/ playgrounds, suitable for pedestrians/cyclists) characteristics | Accounted for by study design: same respondents measured pre & post move. Built environment attributes measured pre & post among movers & non-movers | Socio-demographic, home characteristics, neighborhood perceptions | Change in # walking & bicycling trips not associated with change in objective environment characteristics among movers  Change in # walking trips associated (+) with increase in parks/green/  strips/ playgrounds among non-movers |
| Boone-Heinonen[34] | Quasi-experiment (multiple-group pre/post-test design), US, 1994/95 & 2001/02 | Stratified sample of schools (n=132). Representative sample of US school-based population (grades 11-22yrs of age n=18924) followed into adulthood (18-26yrs of age n=14322; analytic n=12701) | Mover defined if >¼ mile from origin.  Men:  15.5yrs (pre: mean age)  21.9yrs (post: mean age)  Women (50.8%):  15.3yrs (pre: mean age)  21.7yrs (post: mean age) | Self-reported frequency of leisure-time moderate-vigorous activity/wk (MVPA) | Street connectivity w/in 1 km (ratio of observed to max possible alternatives between intersections, pay recreational facilities/10000 population w/in 3 km, public recreational facilities/10000 population w/in 3 km, landscape diversity (Simpson’s diversity index)w/in 1 km, population count w/in 3 km (based on Euclidean distance) | Accounted for by study design: same respondents measured pre & post move. Built environment attributes measured pre & post among movers & non-movers | Sex-stratified, socio-demographics | Change in MVPA frequency associated (-) with increase in landscape diversity (pre to post) among non-movers  Change in MVPA frequency associated (+) with increase in public recreational facilities (pre to post) among women movers  Change in MVPA frequency associated (men + /women -) with increase in pay recreational facilities among movers & non-movers |
| Coogan[35] | Quasi-experiment (single-group pre/post-test design), New York, Chicago, Los Angeles, US, 1995-2001 | Convenient sample of African-American women magazine subscribers (n=20354; n=12790 with 6yrs; n=3206 with 4yrs, &; n=4358 2yrs of follow-up) | Across the 3 locations:  39.2yrs (mean age)  46.9% with ≥16yrs education | Self-reported time/wk in prior year 1) walking for exercise & 2) walking to/from church, store, school, work (7 categories: 0 to ≥10 hrs/wk) | Housing density, land use (% residential & non-residential land use area), connectivity (block size, # 4-way intersections), traffic (length of major roads in buffer), availability of public transit & buses, presence of sidewalks, distance to parks (≥5 acres in area) within 1.6km of respondents household  Only change in housing density was examined longitudinally | Accounted for by study design: same respondents measured pre & post move. Built environment attributes (housing density) measured pre& post testing among movers. | Socio-demographics health behavior, health status, neighborhood characteristics | Change in transport walking associated (-) with decrease in density  Change in exercise walking associated (+/-) with increase in density |
| Evenson[40] | Quasi-experiment (single-group pre/post-test design), Durham, North Carolina, US, 2000-2002 | Randomly selected households w/in 11 census blocks (w/ 2 miles) traversing a proposed rail-to-trails conversion project (n=685 w/baseline; R=47.2%; n=685; w/follow-up; R=63.7%). Time between pre-post testing (trail construction) 19-28mths (analytic n=366) | 64.7% women  42.% ≥50yrs  75.1% ≥16yrs education | Self-reported leisure activity, walking & bicycling, & moderate & vigorous physical activity days & total time/wk & use of trail  Change in physical activity pre-post overall & based on recommended PA from leisure & moderate/ vigorous activity | The new trail extended (by 2.8 miles) an established trail (10 feet wide, paved for pedestrians, cyclists, & other users). The new trail passed by schools, shops, apartments, sub-divisions & includes many access points.  Shortest Euclidean distance to the trail from participants home | Accounted for by study design: same respondents measured pre & post change in the environment | Socio-demographics health status, environmental characteristics, weather | Walking time less likely to increase among those using the trail  Bicycling time more likely to decrease among those using the trail |
| Fitzhugh[43] | Quasi-experiment, (multiple-group pre/post-test design), Knoxville, Tennessee, US, March 2005-March 2007 | Respondents recruited (via direct observation) from 1 intervention & 2 control neighborhoods (matched on education, ethnicity, unemployment, sex, mortgages, median age, household income) | Neighborhood-level demographic characteristics presented (census data).  Intervention neighborhood:  9.3%<high school education  50.2% women  30yrs (median age)  Two control neighborhoods (mean estimates):  9.7%<high school education  53.0% women  39.5yrs (median age) | Direct observation of neighborhood physical activity in pre/post intervention. Pedestrian, cyclists, other forms of physical activity (e.g. skate-boarding) also captured | Neighborhood fitted with an urban greenway/trail, with intent to provide pedestrian-friendly links among residences, businesses, & other public space. New trail was 8ft wide& 2.9 miles long | Partly accounted for by the study design. Pre & post-intervention testing in the same neighborhoods but not necessarily the same respondents | None at the individual level. Neighborhoods matched on several characteristics | Walking & cycling counts increase in intervention & decreased in the control neighborhoods (pre to post)  Pedestrian, cycling, & total physical activity counts were higher in the intervention compared with control neighborhood (pre to post). |
| Lee[36] | Quasi-experiment (single-group pre/post-test design), US, 1988-1993 | Undergraduate males from Harvard University in 1916-1950 (n=3448 with mapped address information in 1988 & 1993) | Not shown | Self-reported daily walking, stair climbing, sports & recreational activities in the past week, frequency & duration. Estimated energy expenditure used as outcome  Mean distance walked/wk also examined | Sprawl index estimated for each participants 1988 & 1993 county. Sprawl index captured attributes related to gross population density, % living in high/low densities, population/mile^2^ of urban land, mean block size & % blocks ≤500 feet on a side.  3.9% (n=135) moved to a more sprawling county; 2.1% (n=73) to a less sprawling county; the remaining (n=3240) stayed in same sprawl | Accounted for by study design: same respondents measured pre & post moving to a new county. | Socio-demographics, health behavior | No change in activity energy expenditure resulting from change in sprawl.  No change in walking distance resulting from change in sprawl. |
| Khattak[56] | Cross-section, 2 neighborhoods (1 conventional , 1 neo-traditional), North Carolina, US | All members (>16yrs of age) of single-family households w/in the two neighborhoods (n=122 conventional, RR=23.6%; n=188 neo-traditional, RR=26.4%) | Conventional neighborhood:  65% male heads of household  47.3yrs (median age) for household head  $100001annual income  Neo-traditional neighborhood:  54% male heads of household  45.9yrs (median age) for household head  $150000 annual income | Self-reported count of walking trips/day at the household level | Compared with neo-traditional neighborhood the conventional neighborhood had: lower gross housing density, lower net single-family housing units, fewer 3 & 4 way intersections, larger block length, no commercial space & no jobs | Statistical adjustment using instrumental variables reflecting indicators of residential choice | Household size, vehicles/ household | # walking trips was associated (+) with the probability of residing in a neo-traditional neighborhood |
| MacBeth[28] | Quasi-experiment (single-group pre/post-test design), Toronto, Canada, 1993-1995 | Respondents (cyclists) recruited via Direct observation along streets designated for instalment of bicycle lanes in downtown (Davenport Rd, Gerrard St., Sherbourne St., Harbord St., St. George St., College St.) | Not shown | Directly measured counts of cyclists on streets with bicycle lanes/day | Street instalment of bicycle lanes which included reconfiguring 4-lane roads to 2-lane roads(plus bicycle lanes), bicycle lane signs & pavement markings, reductions in speed limit to 40km/hr | Partly accounted for by the study design. Pre & post-intervention testing in the same streets but not necessarily the same respondents | None included | Bicycle traffic/day increased in all streets following installation (about 2yrs later) of bicycle lanes (increase of 4-42%, average=23%) |
| Goulias[41] | Quasi-experiment (single-group pre/post-test design)/quasi-longitudinal, Salt Lake City, US, February-October 2007 | Respondents from households in 1 suburban neighborhood (Academy Park) spatially stratified w/in 1 mile of a proposed trail (n=290 households, n=796 respondents). N=117 new residents (post-trail construction also recruited). Analytic sample with pre/1 mth post data n=144 & pre/5 mth post data = 98. N=31 trail users participated in intercept surveys | Presented for entire sample (not intercept sample):  45.1% male  82.5% ≥18yrs  47.8yrs (mean age)  37.8% $40000 annual household income | Self-reported episodes & minutes of total physical activity/day & number of walking & biking trips/day measured before trail constructions, 1-month, & 5 months post construction  Change in activity prior to trail construction among trail users (Wednesday & Saturday) | Neighborhood fitted with a two-way multi-use trail separated from existing roads & sidewalks along an existing canal right-of-way. The trail was adjacent to 2 major schools & created a loop with 2 existing sidewalks | Accounted for by study design: same respondents measured pre & post intervention | Socio-demographics, built environment, day activity data collected | No change in total physical activity (episodes or minutes) or total walking or biking trips 1 mth post trail construction  Total physical activity episodes & total walking trips decreased from pre to 5-mth post trail construction  87% of trail users reported do the same activity prior to the trail construction |
| Boarnet[58] | Cross-section, Central Metropolitan area, Portland Oregon, US, 1994 | Respondents from households recruited for a travel survey (n=8042 individuals recruited, no RR presented) (analytic n=4699-6362) | 51.8% women  23.3% ≥50yrs  21.2% ≤16yrs  $45154 annual household income | Self-reported two-day walking distance | Population density, employment density, retail employment density, pedestrian environment factor (PEF: includes quality of sidewalk infrastructure, slope, characteristics of pedestrian environment), # intersections, distance from city hall, distance from light rail station measured at the smallest geographical scale available (census block groups and transportation analysis zones) | Statistical adjustment using a Heckman selection model to adjust for unmeasured variables (including residential selection) associated with choice to reside in low, medium, or high PEF neighborhood | Socio-demographics, physical disability, day walking measured | Distance walked associated with employment density (+), # intersections (+), distance to city hall (-), distance to light rail (+) adjusting for unmeasured estimate of residential selection (inverse mills ratio, although most were attenuated. Association between population and retail density and walking no longer significant after adjustment. |
| Brown[42] | Quasi-experiment (single-group pre/post-test design) Salt Lake City neighborhood, Utah, US, summer 2005/2006 | Respondents residing within ½ mile of new rail stop recruited via door-to-door visits (n=215; R=47.44%; analytic n=47 with follow-up accelerometer data) | 47% women  41±13.83yrs of age  $24000-43367 annual household income (neighborhood mean)  5±7yrs residing in neighborhood | Accelerometer counted bouts/hr of moderate-intensity activity among non transit riders, new riders, and continuing riders | A new rail stop added between two existing stops. New stop added to a neighborhood with industrial land uses, subsidized apartment, multifamily, & small single-family detached housing, grid street pattern, tree-lined sidewalks, with few walkable destinations beyond convenience stores | Accounted for in the study design: same respondents from same neighborhood measured pre & post intervention. | Socio-demographics | No difference in physical activity among the three ridership groups over time (i.e., pre-post intervention) |
